# A Retrospective Analysis of Malaria Trends in Maksegnit Health Center over the Last Seven Years, Northwest Ethiopia: 2014-2020

**DOI:** 10.1155/2022/5170550

**Published:** 2022-05-24

**Authors:** Tegegne Eshetu, Bedruzeman Muhamed, Merima Awol, Zebie Kassa, Mehabaw Getu, Adane Derso, Aberham Abere, Ayalew Jejaw Zeleke

**Affiliations:** ^1^Department of Medical Parasitology, School of Biomedical and Laboratory Science, College of Medicine and Health Sciences, University of Gondar, P. O. Box: 196, Gondar, Ethiopia; ^2^School of Biomedical and Laboratory Science, College of Medicine and Health Sciences, University of Gondar, P. O. Box: 196, Gondar, Ethiopia

## Abstract

**Background:**

In Ethiopia, despite various public health intervention approaches have been implemented to eliminate malaria, its public health problem remains considerable. There are such numerous studies; however, investigating the trend of malaria infection in various settings is paramount for area-specific evidence-based interventions, evaluating ongoing malaria control programs. Hence, since the trend of malaria infection in Maksegnit has not yet been documented, this study is aimed at assessing the seven-year trend of malaria in Maksegnit Health Center.

**Methods:**

An institutional-based retrospective study was conducted to assess the trend of malaria prevalence over the last seven years (2014-2020) using recorded blood smear reports in the laboratory logbook in Maksegnit Health Center, Northwest Ethiopia.

**Result:**

Over the last seven years, a total of 28217 clinically malaria-suspected individuals were requested for blood film examination at Maksegnit Health Center. Of whom, microscopically confirmed malaria case was found in 4641/28217 (16.4%). A significant seasonal and interannual variation of malaria cases was observed (*P* < 0.001). The highest prevalence was observed in years 2014 (25.5%) and 2020 (25.1%), while the minimum annual prevalence was seen in 2017/18 (6.4%). The month of October (25.5%) had the highest number of malaria cases documented, while February had the least (4.7%). Males and individuals under the age group of 15-45 were the most affected segments of the population. A significant interannual fluctuating prevalence of malaria cases was recorded ranging from 25.5% to 6.4% (*P* < 0.001).

**Conclusion:**

Malaria is still a public health threat in the study area despite significant fluctuating patterns of malaria was observed in the last seven years. In particular, a bounced back trend of malaria from 2018 to 2020 is alarming. Thus, the implementation of ongoing intervention approaches should be reconsidered, and uninterrupted efforts of the concerned bodies are still needed.

## 1. Background

Although malaria is a treatable and preventable parasitic disease, yet, it continues to be a major public health issue that blights the lives of billions of people worldwide [1]. There are five known *Plasmodium* parasites, viz., *Plasmodium falciparum*, *Plasmodium vivax*, *Plasmodium malariae*, *Plasmodium ovale*, and *Plasmodium knowlesi*, that cause malaria in humans through the bite of female *Anopheles* mosquitoes. Two of these *Plasmodium* spp., *P. falciparum* and *P. vivax*, are the most common species that pose the greatest threat in mankind [2]. In 2020, there were an estimated 241 million malaria cases and 627 000 malaria deaths globally. This indicates that malaria cases and deaths in 2020 were increased by 14 million and 69,000, respectively, as compared to 2019 report. Of these, Sub-Saharan Africa took the lion share of the global burden of malaria with an estimated 95% of malaria cases and 96% malaria associated deaths in 2020. Children under the age of five accounted for around 80% of all deaths in the region [3].

In Ethiopia, where nearly three-fourths of the country's landmass is favorable for malaria transmission, more than 60 million people (60% the population) live in malarious areas [4]. Indeed, over the last decade, the morbidity and mortality of malaria has been remarkably reduced following implementation of public health intervention measures including insecticide-treated mosquito nets (ITNs), indoor residual spraying (INR), accurate diagnosis and prompt treatment with artemisinin-based combination therapies (ACT), and intermittent preventive treatment of pregnant women (IPTp) throughout the country. However, recent findings indicate that the incidence of malaria is coming increasingly globally, and Ethiopia has proven to be no exception [5, 6]. In 2020, Ethiopia accounted for 1.8% and 1.5% of all malaria cases and deaths globally, respectively [3]. The morbidity level and transmission status of malaria in most parts of Ethiopia is unstable and show marked seasonal, interannual, and spatial variability [7]. The instability of malaria transmission pattern relay on altitude, rainfall, and variation in socio-demographic risk factors. Its incidence and transmission mainly occur in two major (September–December) following a heavy summer rainfall season and minor (April–June) following short rainy seasons [6, 8–10]. This unstable transmission pattern along with the recent evidence of shifting malaria transmission pattern to the previously unexposed area may be an obstacle for the success of the national plan of eliminating malaria in certain low transmission settings by 2020 and eradicated by 2030.

Furthermore, the seasonal migration of adult male laborers from the study region to the Metema–Humera lowlands, where malaria is highly common, is complicating the malaria elimination campaign, particularly in northwest Ethiopia. The migrant returnees with other aggravating factors in play, the study area renowned in malaria outbreaks at various times, despite there is lack of clear evidence regarding the morbidity and mortality of malaria in Maksegnit. As a result, the goal of this study was to indicate the prevalence and trend of malaria in the study area during the last seven years in order to assess the impact of intervention strategies, because we believed that such analysis of malaria morbidity trends in endemic areas would help to understand the dynamics of malaria transmission. Besides, such information is crucial to evaluate the effectiveness of current intervention, approaches to control disease burden, and mapping evidence-based interventions. Thus, herein, we assessed a seven-year trend of malaria and distribution over sex, age, and season in Maksegnit Health Center, northwest Ethiopia.

## 2. Materials and Methods

### 2.1. Study Area and Period

The study was conducted in Maksegnit Health Center which is located in central Gondar zone, Amhara regional state, located 40 km from Gondar town, Ethiopia. The area is located at latitude, longitude, and altitude of 12.3-13.8° N, 35.3-35.7° E, and 2,220 at sea level. Maksegnit Health Center is the only health institute that provides healthcare services to the community in the town. The local average temperature varies from 15°C to 31°C and is characterized by two main rainy seasons (June-August) peak rainfall season and (March-May) minor rainy season. Based on the local health bureau report, Maksegnit is renowned for a periodic outbreak of malaria cases during the major rainy seasons. The data was retrieved from the laboratory log book between January and February 2021.

### 2.2. Study Design and Population

A health facility-based retrospective study was conducted to determine the trend, prevalence, and distribution of malaria over sex, age, and season over the past seven years in Maksegnit Health Center. The study populations were all malaria-suspected individuals who provided blood samples for blood film microscopy and registered in the lab logbook of the health center.

#### 2.2.1. Inclusion and Exclusion Criteria

Participants who had full recorded data such as age, sex, year, and date of diagnosis were included in the study, whereas individuals who had incomplete recorded information in the logbook were excluded.

### 2.3. Data Collection Tools and Techniques

Prior to extracting participants' data from the laboratory logbook, a data extraction sheet that was specifically designed to address participants' demographic data (age, sex), month, and year of diagnosis was prepared using Microsoft Excel. Then, the variables of interest were transferred from the laboratory logbook to the preprepared data extraction excel sheet. The extracted study variables included clients' sex, age, date of diagnosis, diagnostic tools used, results of investigation, and species of parasite detected. To assure the quality and consistency of the data, data collectors were trained regarding the data extraction tool, variables of interest, and the objective of the study. Besides, every activity of the data extraction and entry process was supervised by the study team members.

### 2.4. Data Processing and Analysis

Prior to any statistical analysis, data were coded and checked for its completeness using epi data software and transferred to SPSS software for statistical analysis. Descriptive statistics was used to show the distribution of malaria transmission in terms of individuals' sex, age, season, and species of parasite detected. Pearson's *χ*^2^ test was carried out to assess the association between dependent variable with independent variables, and *P* value < 0.05 was considered statistically significant. The results were displayed using text, graphs, and tables.

## 3. Results

### 3.1. Annual Prevalence of Malaria in Maksegnit Health Center (2014-2020)

Over the last seven years, a total of 28217 clinically malaria-suspected individuals were requested for blood film examination at Maksegnit Health Center. Of whom, 16311 (57.8%) of the suspected individuals were male. The majority of the study participants (58.4%) were in the age group of 15-45 years. Of the total suspected participants, microscopically confirmed malaria was found in 4641 suspected individuals with an estimated positivity rate of 16.4% (95% CI: 16%-16.9%). On average, 663 microscopically confirmed malaria cases were recorded annually. In all years of investigation, *Plasmodium falciparum* was the predominant malaria parasite identified with an estimated prevalence of 9.8%, followed by *Plasmodium vivax* (5.7%). The remaining 0.8% cases of malaria were *Plasmodium falciparum*-*Plasmodium vivax* mixed infections (Table 1).

### 3.2. Trends of Malaria Cases in Maksegnit Health Center from 2014 to 2020

**Figure 1:**
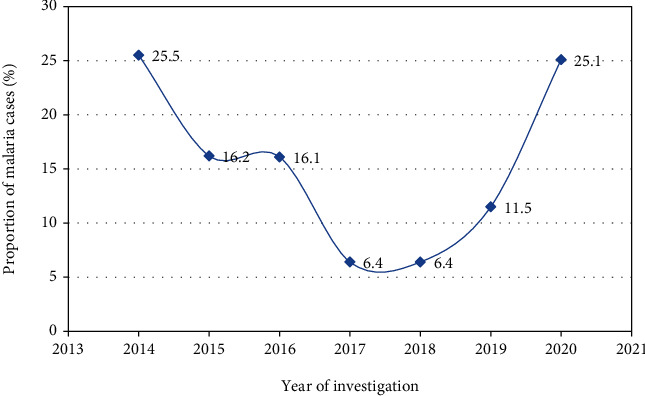
Trends of malaria prevalence in Maksegnit Health Center, northwest Ethiopia (2014-2020).

Over the last seven years, the prevalence of malaria was highly fluctuating across years ranging from 25.5% to 6.4% (*P* < 0.001). The highest prevalence was observed in 2014 (25.5%), followed by 2020 (25.1%). Although the trend of malaria prevalence was observed with a fragile and inconsistent distribution across years, a generally steady declining trend prevalence of malaria was recorded from 2014 to 2017 with an estimated prevalence of 25.4% to 16.4%, respectively. However, constant malaria case had been documented for two consecutive years from 2017 to 2018. On the other hand, a significant increment trend of malaria prevalence was observed from 2018 (6.4%) to 2020 (16.4%) (Figure 1).

### 3.3. Seasonal and Monthly Distribution of Microscopically Confirmed Malaria Cases (2014-2020)

**Figure 2:**
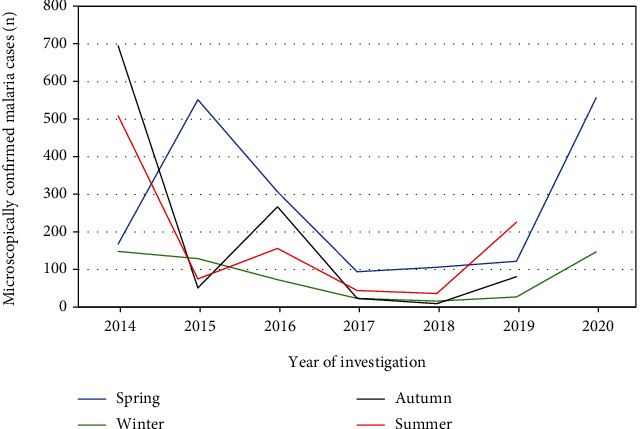
Seasonal distribution of malaria among individuals requested for malaria examination at Maksegnit Health Center from 2014 to 2020 (*n* = 28217).

In the concern of monthly distribution analysis of malaria cases, statistical significant variation of malaria distribution was confirmed across months with a range of 25.5% to 4.7% (*P* < 0.001). The months of October (25.5%), November (22.7%), and May (21.8%) had the highest malaria cases documented, while February had the least (4.7%) (Table 2). In addition, the distribution of confirmed malaria cases showed statistical significant associations with seasons (*χ*^2^ = 387, *P* < 0.001). Autumn had the highest malaria cases (22.3%), followed by spring (15.6%) and summer (15.1%). Winter (December to February) was the season with the least recorded malaria cases (10.1%; *P* < 0.001) (Figure 2).

### 3.4. Distribution of Confirmed Malaria Cases in relation to Sex and Age Groups

**Figure 3:**
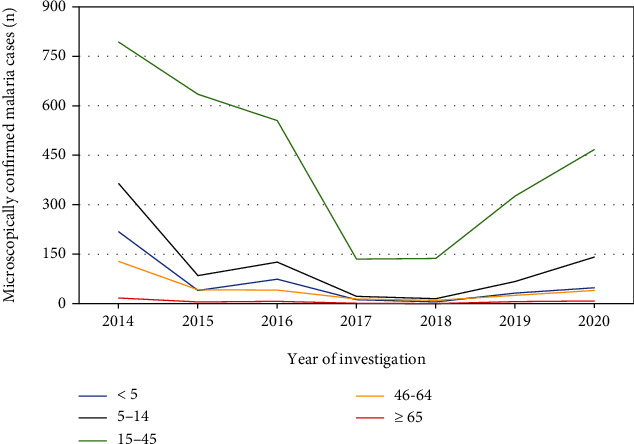
Trend analysis of malaria in relation to participants' age from 2014 to 2020 (*n* = 28217).

The overall prevalence of malaria over the last seven years in Maksegnit Health Center showed a statistical significant variation among different age groups (*χ*^2^ = 215, *P* < 0.001). Of all confirmed malaria cases, individuals within the age group of 15-45 years had the highest malaria cases (10.8%) followed by the age group of 5-14 (2.9%), while individuals whose age ≥ 65 years were identified with the record of the least malaria cases (0.2%). The remaining 429 (1.5%) and 300 (1.1%) confirmed malaria cases were in the age of <5 and 46-64 years, respectively. Moreover, regarding the trend analysis of malaria distribution among age groups, individuals whose ages lie in the range of 15-45 years were identified as the most affected segments of the population across all years (Figure 3).

**Figure 4:**
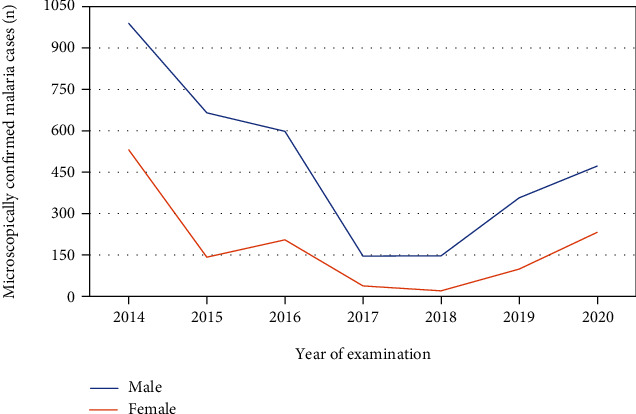
Trend analysis of malaria in relation to participants gender from 2014 to 2020 (*n* = 28217).

Moreover, our findings showed that males were found to be more affected with *Plasmodium* infection than females with a statistical significant level over the last seven years with an estimated prevalence of 12% (3374/28217) and 4.5% (1267/28217), respectively. In addition, males account the majority of malaria cases across all age groups (Figure 4).

## 4. Discussion

Despite significant advances in malaria control over the last two decades, which has increased enthusiasm for the achievement of the WHO-set goal of reducing malaria incidence and mortality by at least 90% by 2030, a worryingly trend of malaria incidence and mortality has been observed in recent years [3, 11, 12]. This alarming trend of malaria is particularly pronounced in Sub-Saharan African countries, including Ethiopia. Since malaria is not a year-round phenomenon in Ethiopia, the intensity of transmission and distribution varies across seasons and years, depending on the nature of the rainy season and local commitment of implementing intervention measures [13]. As a result, we assessed the transmission dynamics of malaria across different seasons to specifically identify malaria peak seasons and years in order to evaluate the success of ongoing intervention strategies in the surrounding community using a recorded data at Maksegnit Health Center for the last seven years.

According to the review of the health center's recorded blood smear data from 2014 to 2020, a total of 4641 (16.4 percent) participants had microscopically confirmed malaria cases from 28217 clinically malaria-suspected individuals. This figure indicates that malaria is still a major public health issue in the study area. Malaria cases reported in 2020, in particular, with an estimated annual prevalence of 25.1%, are concerning and may call into question ongoing public health intervention efforts. Studies conducted in Dembecha, west Gojjam zone of Amhara regional state and Jardega Jarte district, Horo guduru Wollega zone of Oromia regional state, Ethiopia [14, 15], support the overall finding of malaria prevalence over the last seven years in Maksegnit Health Center. However, our finding is lower than other retrospective studies conducted in Adi Arkay [16], in selected zones of Amhara regional state [17], Bale zone [18], Kersa district of Oromia region [19], Gorgora [20], Jimma [21], Kola Diba [22], Guba [7], Tselemt [23], and Wolaita [24], with a positivity rate ranging from 21.8 to 66.7%. On the contrary, studies conducted in Arsi Negelle (11.45%), Ataye (8.4%), Bahrdar (5%), Bichena (9.28%), Halaba (9.5%), Woreta (5.6%), Libokemkim (10.9%), Wolketie (8.56%), and Kombolcha (7.52%) reported lower malaria cases [25–33] when compared with our finding.

Local community and leaders' commitment to implementing the recommended public health intervention measures laboratory personnel skills in malaria microscopy, geographical location of the study setting, applied diagnostic tools, study period, and accessibility of control measures differed from one place to another which could all be the possible reasons for the discrepancies. For instance, regarding diagnostic tool variation, a study conducted in bale Zone [18], Ethiopia, and in selected Zone of Amhara region [17] used a combined malaria microscopy and RDT result to estimate the overall prevalence of malaria in their respective area, whereas our study used only malaria microscopy report. As a result of the differences in diagnostic sensitivity between the two techniques, the superiority of their report over the current report could be due to the overestimation effect of malaria RDT, which could be the plausible reason for the inconsistency of the result.

In addition, a significant interannual fluctuated prevalence of malaria was observed with a maximum and minimum annual prevalence of malaria documented in 2014 (25.4%) and 2017/18 (6.4%), respectively. In this study, a declined trend of malaria cases was seen from 2014 (25.5%) to 2018 (6.4%). This observed significant improvement may be achieved by the application of the designed public health intervention measures in the community due to the severity of the disease burden at that time. However, significant reverted cases of malaria from 2018 (6.4%) to 2020 (25.1%) were observed followed by a constant case of malaria that had been documented for two successive years 2017/18. This reverted trend of malaria is consistent with the recent WHO reports of malaria [3]. The rise in drug and insecticide resistance, climatic change, and asymptomatic infections as a silent transmission of malaria could all be factors for the recent resurgence of the disease. Furthermore, the recent impact of COVID-19 on malaria control and elimination efforts may also be a possible factor for the rebounce back of malaria trend since 2018. This shocking trend of malaria recorded recently raises many concerns on the ongoing prevention and control efforts in the community. This finding could be suggestive of the continued occurrence of high malaria burden in the community which may need collaborative efforts and careful attention from the government and other stakeholders to minimize the public health problems in the surrounding community.

In the concern of confirmed cases of malaria distribution among the different age groups, greater numbers of malaria cases were observed among individuals whose ages ranged from 15 to 45 years. This more pronounced burden among the productive segment of the population made the impact of malaria more burning and concerning issues in the community, because this age group of the population are under the time of carrying different responsibilities of their family, society, and other else. The disproportionate share of malaria in this segment of the population might be due to their higher chance of mobilization to different areas and outdoor activities. Moreover, since agriculture is the major source of income for the majority of residents in the town and surrounding environments, the chances of exposing to different outdoor activities and migrating to malaria-endemic farmlands are high. Due to these possible reasons, individuals in this age group are more prone to malaria. In addition, in our findings, males were the most affected segments of the population than females. It may be related to increased engagement of males in many outdoor tasks, farming activities, and cattle keeping occasions when compared with females in the community.

Our findings also assured that the occurrence of malaria in every month of the year despite the burden of morbidity in each month is significantly varied. The months of October, November, and May had the highest malaria cases documented, while February had the least cases of malaria in almost all year round. Regarding the seasonal distribution of malaria in our findings, autumn (September to November) had the highest malaria cases followed by spring (March to May), which is supported by the fact that in Ethiopia, malaria transmission hit the highest level from September to December (following the main rainy season) and April to May (after the minor rainy season of the country) [34]. These peaks of malaria transmission seasons coincide with the major harvesting time throughout the country, as a result of the overlapping period of the major harvesting time and peak malaria transmission seasons suitable for the aggravation of malaria transmission in the study area. During harvesting time, outdoor activities and frequency of contact with cattle are higher than other times. As a result, such outdoor activities increase the risk of malaria due to the incidence of outdoor mosquito biting is increased following the vector's host-seeking behavior being changed as recent evidence indicated [35]. Moreover, having frequent contact and proximity with cattle increases the probability of biting with mosquitoes malaria vector and has a behavior of attracted by cattle [36]. In this study, showing the burden of malaria cases over the last seven years with a large sample size considered as a positive side unable to address the bottlenecked factors which trigger and reaggravating the severity of malaria in recent times is the main limitation of the study.

## 5. Conclusion

In this study, although the burden of malaria cases declined from 2014 to 2018, a reverted and sharply increment trend of malaria cases was observed from 2018 to 2020. This indicates that still malaria remains the major public health problem in the community particularly in the age group of 15-45 years which is the productive segment of the population. This implies there is something that bottlenecks the effectiveness of the ongoing control efforts. Thus, the ongoing malaria prevention and control strategies should be reconsidered, and the respective stakeholders/bodies should take a strict commitment to the implementation of the designed control and prevention efforts. The main causes which returned the burden of malaria burden back to its alarming status should be investigated by the local health bureau or health extension workers or by any other concerned body.

## Figures and Tables

**Table 1:** Microscopically confirmed malaria cases among individuals who visited Maksegnit Health Center from 2014 to 2020 (*n* = 28217).

Year	Total number of blood films examined	Microscopically confirmed malaria cases, *n* (%)	*Pf*	*Pv*	Mixed (%)
2014	5972	1520 (25.4)	982 (16.4)	537 (9.0)	11 (0.2)
2015	4971	807 (16.2)	422 (8.5)	311 (6.3)	74 (1.5)
2016	4987	803 (16.1)	433 (8.7)	272 (5.5)	85 (1.7)
2017	2890	184 (6.4)	110 (3.8)	63 (2,2)	7 (0.2)
2018	2624	167 (6.4)	111 (4.2)	46 (1.8)	5 (0.2)
2019	3970	456 (11.5)	244 (6.1)	171 (4.3)	35 (0.9)
2020	2803	704 (25.1)	473 (16.9)	221 (7.9)	9 (0.3)
Total	28217	4641 (16.4)	2775 (9.8)	1621 (5.7)	226 (0.8)

*Pf*: *Plasmodium falciparum*; *Pv*: *Plasmodium vivax.*

**Table 2:** Monthly distribution of malaria among individuals requested for malaria examination at Maksegnit Health Center from 2014 to 2020 (*n* = 28217).

Months of examination		Microscopic examination result	
	Total number of suspected individuals (*N*)	Positive *N* (%)	Negative *N* (%)
January	1763	120 (6.8)	1643 (93.2)
February	1364	64 (4.7)	1300 (95.3)
March	2187	239 (10.9)	1948 (89.1)
April	2384	315 (13.2)	2069 (86.8)
May	2624	572 (21.8)	2052 (78.2)
June	2323	374 (16.1)	1949 (83.9)
July	2110	250 (11.8)	1860 (88.2)
August	2478	422 (17)	2056 (83)
September	2426	434 (18.1)	1988 (81.9)
October	2722	695 (25.5)	2027 (74.5)
November	3403	773 (22.7)	2630 (77.3)
December	2433	379 (15.6%)	2054 (84.4%)

## Data Availability

The data generated or analyzed during this study is included in this manuscript. Other data will be available from the corresponding author upon request.

## Abbreviations

WHO: World Health Organization
SPSS: Statistical Software for Social Science
*Pf*: *Plasmodium falciparum*
*PV*: *Plasmodium vivax*.
